# Eating Behavior in Menopausal Women: From Molecular to Clinical Aspects—What Changes?

**DOI:** 10.3390/ijms27198538

**Published:** 2026-09-24

**Authors:** Sandro La Vignera, Aldo E. Calogero, Rossella Cannarella, Andrea Crafa, Federica Barbagallo, Rosita A. Condorelli

**Affiliations:** Department of Clinical and Experimental Medicine, University of Catani a, Via Santa Sofia 78, 95123 Catania, Italy

**Keywords:** menopause, eating behavior, estrogen receptors, hypothalamic neuropeptides, leptin, ghrelin, visceral adiposity, metabolic syndrome, gut microbiota, hormone replacement therapy

## Abstract

The menopausal transition represents a critical period in women’s lives characterized by profound hormonal, metabolic, and behavioral changes. Estrogen deficiency during menopause triggers complex alterations in eating behavior through multiple molecular mechanisms involving hypothalamic neuropeptide systems, neurotransmitter pathways, and peripheral hormonal signals. This narrative review examines the molecular basis of eating behavior changes in menopausal women, integrating evidence from basic science and clinical research. At the molecular level, declining estradiol disrupts the hypothalamic regulation of appetite through estrogen receptors ERα and ERβ, leading to dysregulation of key neuropeptides including neuropeptide Y (NPY), agouti-related peptide (AgRP), and proopiomelanocortin (POMC). Peripheral signals such as leptin, ghrelin, and insulin show altered secretion and sensitivity patterns. Clinically, postmenopausal women experience increased appetite, heightened food reward sensitivity, carbohydrate cravings, and elevated risk of binge eating behaviors. These changes contribute to weight gain averaging 2–3 kg during the menopausal transition, with preferential visceral fat accumulation increasing metabolic syndrome risk. Body composition shifts include 44% increases in visceral adiposity and concurrent sarcopenia. Emerging evidence highlights the role of gut microbiota alterations in mediating metabolic dysfunction. Therapeutic strategies including hormone replacement therapy, targeted nutritional interventions, and lifestyle modifications show promise in mitigating these changes. Understanding the molecular-to-clinical continuum of eating behavior changes in menopause is essential for developing evidence-based preventive and therapeutic approaches to improve metabolic health and quality of life in postmenopausal women.

## 1. Introduction

Menopause, defined as the permanent cessation of menstruation resulting from loss of ovarian follicular activity, represents a universal biological transition affecting all women who reach midlife. The menopausal transition, or perimenopause, typically begins in the fifth decade of life and is characterized by progressive decline in ovarian estrogen production, culminating in the final menstrual period [1,2]. This hormonal shift triggers a cascade of physiological changes affecting multiple organ systems, with particularly profound effects on energy metabolism, body composition, and eating behavior [3,4].

Epidemiological evidence demonstrates that the menopausal transition is associated with significant metabolic alterations. Postmenopausal women experience an average weight gain of 2–3 kg, with visceral fat mass increasing by 44% and gynoid fat by 32% [5,6]. More concerning than absolute weight gain is the redistribution of adipose tissue from subcutaneous to visceral depots, a pattern strongly associated with insulin resistance, dyslipidemia, hypertension, and increased cardiovascular risk [1,7]. The prevalence of metabolic syndrome increases dramatically during menopause, occurring three times more frequently in postmenopausal compared to premenopausal women [5]. These metabolic disturbances contribute to a 26–30% increase in coronary heart disease mortality among postmenopausal women.

Central to these metabolic changes are alterations in eating behavior. Clinical observations reveal that postmenopausal women exhibit increased hunger, enhanced desire to eat, elevated prospective food consumption, and decreased fasting fullness [3]. Specific changes include heightened preferences for high-fat and high-sugar foods, increased starch intake, and altered macronutrient selection patterns [8,9]. Emerging evidence also suggests elevated risk of disordered eating behaviors, including binge eating during perimenopause and restrictive eating patterns in postmenopause [10,11].

The molecular mechanisms underlying these behavioral changes involve complex interactions between declining estrogen levels and central nervous system pathways regulating appetite and energy homeostasis. Estrogens exert profound effects on hypothalamic circuits through estrogen receptors ERα and ERβ, modulating the activity of key neuropeptide systems including neuropeptide Y (NPY), agouti-related peptide (AgRP), proopiomelanocortin (POMC), and cocaine- and amphetamine-regulated transcripts (CART) [5,6,12]. Additionally, estrogens influence peripheral hormonal signals such as leptin and ghrelin, as well as neurotransmitter systems including serotonin and dopamine, all of which contribute to appetite regulation and food reward processing [13,14].

Despite growing recognition of eating behavior changes during menopause, the molecular-to-clinical continuum remains incompletely characterized. Understanding the mechanistic basis of these changes is essential for developing targeted therapeutic interventions to prevent or mitigate the metabolic consequences of menopause. This narrative review synthesizes current evidence on the molecular mechanisms, clinical manifestations, and therapeutic approaches related to eating behavior changes in menopausal women (summarized in Figure 1), with the goal of providing a comprehensive framework for researchers and clinicians addressing this important aspect of women’s health.

This narrative review was conducted through a systematic search of the PubMed, Scopus, and Web of Science databases, covering publications from 2000 to 2025. Search terms were combined using Boolean operators and included: ‘menopause,’ ‘postmenopause,’ ‘perimenopause,’ ‘eating behavior,’ ‘food intake,’ ‘appetite regulation,’ ‘body composition,’ ‘metabolic syndrome,’ ‘estrogen,’ ‘leptin,’ ‘ghrelin,’ ‘gut microbiota,’ and ‘nutritional intervention.’ Articles published in English were included. Both original research articles and systematic reviews or meta-analyses were considered eligible when addressing clinical, molecular, or therapeutic aspects of eating behavior in menopausal or postmenopausal women. Case reports, editorials, and conference abstracts were excluded. Reference lists of selected articles were manually screened to identify additional relevant sources.

## 2. Molecular Mechanisms of Estrogen Action in Appetite Regulation

### 2.1. Estrogen Receptors and Hypothalamic Signaling

Estrogens regulate energy homeostasis primarily through actions in the central nervous system, particularly within hypothalamic nuclei that control appetite and energy expenditure. The biological effects of estradiol (E2), the predominant circulating estrogen in premenopausal women, are mediated by two classical nuclear estrogen receptors, ERα and ERβ, which function as ligand-dependent transcription factors [4]. Upon E2 binding, these receptors undergo dimerization and interact with estrogen response elements (ERE) in target gene promoters, thereby modulating gene expression [4]. Additionally, ERα can act through rapid, non-genomic mechanisms at the cell membrane and in the cytoplasm, enabling short-term modulation of neuronal activity [4,15].

ERα plays the predominant role in metabolic regulation, with abundant expression in key hypothalamic nuclei including the arcuate nucleus (ARC), ventromedial hypothalamus (VMH), and dorsomedial hypothalamus (DMH) [15]. Genetic studies demonstrate that ERα knockout mice recapitulate most metabolic effects of ovariectomy-induced estrogen deficiency, including hyperphagia (excessive food intake beyond satiety needs), reduced energy expenditure, increased body weight, and visceral adiposity [4,16]. Conversely, ERβ appears to play a more specialized role, particularly in peripheral fat deposition and adipose tissue metabolism [15,17].

The hypothalamic actions of estrogen involve multiple signaling pathways beyond classical genomic mechanisms. Estrogen modulates neuronal excitability through G-protein-coupled membrane estrogen receptors (Gq-mER), activating intracellular signaling cascades including phosphoinositide 3-kinase (PI3K), phospholipase C (PLC), and mitogen-activated protein kinase (MAPK) pathways [15]. These rapid signaling events alter the electrical activity of appetite-regulating neurons, providing a mechanism for acute estrogen effects on feeding behavior [15].

In the arcuate nucleus, ERα directly influences the activity of two critical neuronal populations with opposing effects on appetite: anorexigenic POMC neurons and orexigenic NPY/AgRP neurons [5,6,12]. Estradiol enhances POMC neuronal activity while suppressing NPY/AgRP neuronal activity, thereby promoting satiety and reducing food intake [5,12]. The molecular basis of these effects involves both transcriptional regulation of neuropeptide genes and rapid modulation of neuronal firing rates through membrane-initiated signaling [15]. Recent evidence highlights the importance of estrogen receptor signaling in tanycytes, specialized hypothalamic cells lining the third ventricle that regulate metabolic homeostasis [18].

The decline in circulating estradiol during menopause disrupts these finely tuned hypothalamic regulatory mechanisms. Estrogen deficiency leads to decreased ERα signaling in key metabolic neurons, resulting in reduced POMC activity, increased NPY/AgRP activity, and ultimately increased appetite and reduced energy expenditure [5,6]. This molecular framework provides the foundation for understanding the clinical manifestations of altered eating behavior in postmenopausal women.

### 2.2. Neuropeptide Systems: NPY, AgRP, and POMC

The hypothalamic neuropeptide systems represent the primary effectors through which estrogen regulates appetite and energy balance, integrating peripheral metabolic signals with central neural circuits to control feeding behavior, energy expenditure, and body weight homeostasis [3,6].

Neuropeptide Y (NPY) is one of the most potent orexigenic (appetite-stimulating) peptides in the mammalian brain. NPY neurons are concentrated in the arcuate nucleus and project to multiple hypothalamic and extra-hypothalamic regions involved in feeding control [3]. Estrogens exert inhibitory effects on NPY expression and release, with estradiol decreasing NPY mRNA levels in the hypothalamic paraventricular nucleus (PVN) [6]. During menopause, estrogen deficiency leads to increased hypothalamic NPY expression, contributing to hyperphagia and weight gain [16].

Agouti-related peptide (AgRP) is co-expressed with NPY in arcuate nucleus neurons and functions as a potent orexigenic signal. AgRP acts as an inverse agonist at melanocortin-3 and melanocortin-4 receptors (MC3R and MC4R), blocking the anorexigenic effects of α-melanocyte-stimulating hormone (α-MSH) [3]. Estrogen deficiency increases AgRP expression, and this upregulation contributes to increased food intake and decreased physical activity in ovariectomized animals [16]. The coordinate regulation of NPY and AgRP by estrogen depends on the ratio of ERα to ERβ in hypothalamic neurons [12].

Proopiomelanocortin (POMC) neurons represent the major anorexigenic neuronal population in the arcuate nucleus. POMC is cleaved to produce α-MSH, which activates melanocortin receptors to suppress appetite and increase energy expenditure [5,12]. Estradiol directly stimulates POMC neuronal activity through ERα, enhancing their anorexigenic function at the molecular level by increasing POMC mRNA expression and enhancing neuronal electrical excitability [15]. During menopause, reduced estrogen signaling decreases POMC activity, contributing to increased appetite and reduced satiety [5,6]. Additional neuropeptides influenced by estrogen include cocaine- and amphetamine-regulated transcript (CART), which exerts anorexigenic effects, and galanin, which stimulates fat intake and increases during estrogen deficiency [9,19].

### 2.3. Peripheral Hormonal Signals: Leptin and Ghrelin

Peripheral hormones provide critical feedback signals to the hypothalamus regarding energy status, and their interactions with estrogen signaling are essential for maintaining metabolic homeostasis. Leptin, secreted by adipose tissue in proportion to fat mass, acts as a key satiety signal by inhibiting NPY/AgRP neurons and activating POMC/CART neurons, thereby suppressing appetite and increasing energy expenditure [3,12]. Estrogens potentiate leptin action through multiple mechanisms: increasing leptin receptor expression in the hypothalamus, enhancing leptin receptor sensitivity, and facilitating leptin signaling pathways [5,6].

During menopause, despite elevated leptin levels associated with increased adiposity, central leptin resistance develops, reducing the hormone’s anorexigenic efficacy [9,12]. This leptin resistance results from decreased estrogen-mediated sensitization of leptin signaling pathways [12]. The dysregulation of kisspeptin and leptin as anorexigenic agents plays a significant role in obesity development in postmenopausal women [12].

Ghrelin, produced primarily by the stomach, is the only known orexigenic peripheral hormone. Ghrelin levels rise before meals and decline postprandially, stimulating appetite and promoting food intake through activation of NPY/AgRP neurons [3,20]. Estradiol acts as an antagonist of ghrelin’s orexigenic effects, and the decline in estradiol during menopause may lead to enhanced ghrelin sensitivity [20]. Postmenopausal women show altered postprandial ghrelin dynamics—with greater decreases immediately after food intake and faster re-increases after 60 min—suggesting heightened ghrelin sensitivity [20]. The interaction between estrogen, leptin, and ghrelin creates a complex regulatory network that is disrupted during menopause, leading to increased appetite and food intake [3,6].

### 2.4. Neurotransmitter Pathways: Serotonin and Dopamine

Beyond neuropeptide systems, classical neurotransmitters play important roles in appetite regulation and food reward processing, with estrogen modulating their synthesis, release, and receptor expression. Serotonin (5-HT) is a key neurotransmitter in hunger and satiety regulation. Greater serotonin activity in the hypothalamus is associated with reduced food intake, particularly decreasing consumption of high-fat foods [21]. Estrogens exert anorexigenic effects by increasing serotonin production in the central nervous system and enhancing satiety [13,19]. During menopause, serotonin levels decline in association with decreased estrogen; a significant negative correlation exists between body mass index (BMI) and serum serotonin levels in overweight postmenopausal women (r = −0.5241, *p* < 0.05) [13].

Dopamine plays a critical role in food reward processing and hedonic eating. Dopaminergic signaling in the mesolimbic reward system—particularly in the nucleus accumbens and ventral tegmental area—mediates the pleasurable aspects of food consumption and influences food-seeking behavior [14]. Estrogens modulate striatal dopaminergic processing of flavor hedonics and prefrontal cortex cognitive inhibitory controls over eating [14]. Decreased dopamine activity following estrogen deficiency may contribute to hyperphagia, particularly for high-fat and high-sugar foods [21]. Melatonin, another estrogen-regulated molecule, shows a highly significant negative correlation with BMI (r = −0.87632, *p* < 0.001) in postmenopausal women, suggesting additional hormonal contributions to metabolic dysfunction [13]. The key molecular and hormonal mediators and their effects on eating behavior during the menopausal transition are summarized in Table 1.

## 3. Changes in Eating Behavior During the Menopausal Transition

### 3.1. Appetite and Satiety Alterations

The menopausal transition is characterized by fundamental changes in appetite regulation and satiety signaling. Clinical studies demonstrate that postmenopausal women experience increased hunger, enhanced desire to eat, elevated prospective food consumption, and decreased fasting fullness compared to premenopausal women [3]. This hyperphagia results from the combined effects of reduced estrogen-mediated satiety signaling, increased orexigenic neuropeptide activity, leptin resistance, and enhanced ghrelin sensitivity [3,6,16]. Satiety mechanisms are also compromised, as the reduced activity of POMC neurons and decreased serotonergic tone diminish the brain’s ability to generate and maintain feelings of fullness after eating [5,13]. Modifications in appetite preferences contribute to overconsumption, with 60% of postmenopausal women developing central body obesity.

### 3.2. Food Reward and Hedonic Eating

Beyond homeostatic appetite regulation, the menopausal transition affects hedonic aspects of eating. Estrogen deficiency alters dopaminergic signaling in reward circuits, potentially increasing the motivational drive to consume palatable, energy-dense foods [14,21]. Estrogens modulate striatal dopaminergic processing of flavor hedonics and prefrontal cortex cognitive inhibitory controls over eating [14]. The decline in estrogen during menopause may reduce cognitive control over food intake while simultaneously enhancing the rewarding properties of palatable foods, creating a ‘double hit’ that promotes overconsumption [14]. Emotional eating—consuming food in response to negative emotions rather than physiological hunger—is particularly common in menopausal women and is linked to weight gain, increased BMI, and obesity [22].

### 3.3. Macronutrient Preferences and Cravings

The menopausal transition is associated with specific changes in macronutrient preferences and food cravings. Postmenopausal women exhibit increased starch intake and heightened preferences for carbohydrate-rich foods [8,9]. This carbohydrate craving may reflect attempts to compensate for reduced serotonin levels, as carbohydrate consumption can transiently increase brain serotonin synthesis [13]. The preference for fat-rich diets also increases during menopause, as an increase in galanin levels at the expense of NPY enhances preference for dietary fats [9]. Dietary intake patterns in postmenopausal women are characterized by low fiber, calcium, vitamin D, and vegetable fats, but excessive animal protein, simple carbohydrates, and saturated fats [9]. Menopause is also associated with lower olfactory sensitivity, which may influence food preferences and intake [7].

### 3.4. Disordered Eating Behaviors

Emerging evidence suggests that the menopausal transition is associated with increased risk of disordered eating behaviors. A systematic review found potentially higher levels of binge eating during the perimenopausal stage, while restrictive eating behaviors appeared more common during postmenopause, with strong positive associations between menopausal symptoms and disordered eating [11,23]. Binge eating risk during midlife and the menopausal transition is linked to sensitivity to ovarian hormones, with fluctuating and ultimately declining estrogen levels during perimenopause triggering binge eating episodes in susceptible women [10]. Estrogens stimulate serotonin neurons to inhibit binge-like eating, and estrogen deficiency may remove this protective effect. Women with a history of anorexia nervosa show different hormonal profiles during menopause, including increased total ghrelin levels [20]. Emotional eating, prevalent during menopause, is linked to poor sleep quality, chronic stress, and hypothalamic–pituitary–adrenal axis dysregulation involving cortisol [22].

## 4. Body Composition Changes and Metabolic Consequences

### 4.1. Weight Gain and Fat Mass Accumulation

Weight gain during the menopausal transition is a near-universal phenomenon, with women experiencing an average increase of 2–3 kg [3,17]. However, the weight gain associated with menopause is not simply a consequence of aging but is specifically attributable to estrogen deficiency, as demonstrated by prevention studies using hormone replacement therapy [14,17]. Menopause is associated with a 17% increase in fat mass, with percent body fat potentially 30% higher in postmenopausal women compared to premenopausal women [19,22]. Resting energy expenditure (REE) declines by approximately 420 kcal/day in postmenopausal women compared with premenopausal women, reflecting decreased estrogen-mediated stimulation of thermogenesis, reduced physical activity, and loss of lean muscle mass [14,24].

### 4.2. Visceral Adiposity and Metabolic Syndrome

More concerning than absolute weight gain is the redistribution of adipose tissue during menopause. Postmenopausal women experience a shift from gynoid (subcutaneous, lower body) to android (visceral, abdominal) fat distribution [6,12]. Visceral fat mass increases by 44% and gynoid fat by 32% during menopause [5,6]. Postmenopausal women exhibit a 4.88-fold higher risk of abdominal obesity compared to premenopausal women [5,6]. The prevalence of metabolic syndrome—characterized by abdominal obesity, dyslipidemia, hypertension, and insulin resistance—increases dramatically during menopause, occurring three times more often in postmenopausal women [5]. This contributes to a 26–30% increase in coronary heart disease mortality. Insulin resistance is a central feature of menopausal metabolic dysfunction: postmenopausal women show decreased fasting glucose control and increased area under the insulin curve after oral glucose tolerance testing [25].

### 4.3. Sarcopenia and Lean Mass Loss

While fat mass increases during menopause, lean body mass concurrently decreases, a phenomenon known as sarcopenia. This loss of muscle mass contributes to the decline in resting energy expenditure and further exacerbates metabolic dysfunction [14]. The combination of increased fat mass and decreased lean mass—termed sarcopenic obesity—is particularly detrimental to metabolic health and physical function. Hormone replacement therapy has been shown to preserve fat-free mass in younger postmenopausal women, highlighting the role of estrogen in maintaining lean tissue [14]. Additionally, HRT reduces abdominal fat and increases lean mass, demonstrating the potential for hormonal interventions to favorably modify body composition [17].

### 4.4. Lipedema

Lipedema is a chronic disorder characterized by bilateral symmetric accumulation of subcutaneous adipose tissue in the lower limbs, typically sparing the feet, with a marked hormonal dependency. Although it commonly manifests at puberty or during pregnancy, the menopausal transition represents a recognized trigger for disease worsening. Estrogen withdrawal accelerates pathological adipose tissue deposition and the associated inflammatory state in affected women, making menopause a particularly critical period for lipedema progression. Unlike general menopausal weight gain, lipedema-associated adiposity is characteristically resistant to conventional dietary restriction and exercise interventions. Management strategies include compression therapy, manual lymphatic drainage, and liposuction, which remain the most effective treatment options. Clinicians evaluating postmenopausal women with disproportionate lower-body adiposity should distinguish lipedema from general menopausal adiposity, as the therapeutic approach differs substantially.

## 5. The Role of Gut Microbiota in Menopausal Metabolic Changes

Emerging evidence highlights the gut microbiota as an important mediator of metabolic changes during menopause. The gut microbiome undergoes significant alterations in response to estrogen deficiency, and these changes contribute to weight gain, metabolic dysfunction, and inflammation in postmenopausal women [18]. Menopause is associated with altered gut microbiome composition, with shifts in bacterial diversity and abundance that correlate with metabolic parameters. Visceral adiposity in postmenopausal women is associated with a pro-inflammatory gut microbiome and immunogenic metabolic endotoxemia. The estrogen–gut microbiota axis, termed the ‘estrobolome’, plays a critical role in modulating estrogen metabolism and bioavailability, creating a bidirectional relationship between hormonal status and microbial composition.

The mechanisms linking gut microbiota alterations to metabolic dysfunction in menopause are multifactorial. Dysbiosis can increase intestinal permeability, leading to translocation of bacterial lipopolysaccharide (LPS) and systemic inflammation. Changes in microbial metabolite production—including short-chain fatty acids, bile acids, and trimethylamine N-oxide (TMAO)—influence host metabolism, appetite regulation, and energy harvest from the diet. The gut–brain axis provides another mechanism through which microbiota alterations may influence eating behavior: gut microbes produce neurotransmitter precursors that can influence central appetite regulation, mood, and food reward processing. Animal studies demonstrate that synbiotic interventions combining prebiotics and probiotics showed beneficial effects on metabolic parameters in estrogen-deficient animal models, suggesting potential therapeutic applications for microbiome-targeted strategies in menopausal women.

## 6. Clinical Implications and Therapeutic Strategies

### 6.1. Hormone Replacement Therapy

Hormone replacement therapy (HRT) represents the most direct approach to addressing estrogen deficiency-related metabolic changes during menopause. Clinical evidence demonstrates that HRT effectively prevents or reverses many adverse metabolic consequences of menopause, including weight gain, visceral fat accumulation, and insulin resistance [4,14,17]. HRT reduces abdominal fat and increases lean mass in postmenopausal women [17]. Estrogenic hormone therapy reduces fat mass, improves bone mineral density, and preserves fat-free mass in younger postmenopausal women [14]. Importantly, clinical studies confirm that HRT does not cause weight gain, contrary to popular misconceptions [26]. The effects of HRT on eating behavior are mediated through restoration of estrogen receptor signaling in hypothalamic appetite-regulating circuits, preventing or reversing the hyperphagia and reduced energy expenditure associated with menopause [4]. Current guidelines recommend individualized risk–benefit assessment and preferential use in younger postmenopausal women within 10 years of menopause onset (‘window of opportunity’ hypothesis).

### 6.2. Nutritional Interventions

Dietary modification represents a cornerstone of metabolic management during menopause. A comprehensive dietary modification in hyperandrogenic postmenopausal women resulted in decreased body weight (4.06 kg versus 0.54 kg in controls), reduced waist–hip ratio, lower total cholesterol, decreased fasting glucose, and improved insulin sensitivity [25]. Specific nutritional strategies include: macronutrient optimization with increased dietary fiber and adequate protein to preserve lean muscle mass and support satiety; phytoestrogen supplementation (soy isoflavones) to reduce waist–hip ratio and visceral fat [17]; adequate micronutrient intake for calcium, vitamin D, and other metabolically essential nutrients [9]; and adherence to Mediterranean-style dietary patterns, which address multiple aspects of menopausal metabolic dysfunction simultaneously. The ketogenic diet (KD), characterized by very low carbohydrate intake (<50 g/day), may represent a potential nutritional option in postmenopausal women. By inducing nutritional ketosis, the KD may promote fat oxidation and modulate hypothalamic appetite-regulating circuits via ketone bodies, potentially improving insulin sensitivity and reducing visceral adiposity and triglycerides; however, menopause-specific clinical evidence remains limited and long-term safety data are insufficient—future randomized controlled trials specifically in postmenopausal women are warranted before routine recommendation. Regarding nutritional supplementation, omega-3 polyunsaturated fatty acids—in particular eicosapentaenoic acid (EPA) and docosahexaenoic acid (DHA) derived from fish oil—exert anti-inflammatory effects through the biosynthesis of specialized pro-resolving mediators (SPMs), including resolvins and protectins [15]. Preliminary evidence suggests this anti-inflammatory action may be particularly beneficial in the context of menopausal adipose tissue inflammation and metabolic dysfunction, although menopause-specific RCT data remain limited. Creatine monohydrate supplementation, by increasing phosphocreatine availability and optimizing ATP resynthesis, may support muscle protein synthesis especially when combined with resistance training, offering a potential strategy to counteract sarcopenia in postmenopausal women; however, menopause-specific clinical evidence remains limited and further targeted trials are needed.

### 6.3. Lifestyle Modifications

Comprehensive lifestyle modifications are essential for managing metabolic changes during menopause. Regular physical activity, including both aerobic and resistance training, helps preserve lean muscle mass, reduce visceral adiposity, improve insulin sensitivity, and maintain energy expenditure [19]. Mind–body interventions such as Tai Chi and Qigong have shown benefits for body composition, sleep quality, and emotional eating in midlife and older women [22]. Addressing sleep disturbances through behavioral interventions can improve metabolic outcomes, given that poor sleep dysregulates appetite hormones including leptin and ghrelin [22]. Stress reduction techniques—mindfulness, meditation, cognitive-behavioral approaches—may reduce emotional eating and improve metabolic health by mitigating hypothalamic–pituitary–adrenal axis dysregulation [22]. At the molecular level, resistance training activates the mTOR signaling pathway, promoting muscle protein synthesis, while aerobic exercise upregulates serotonin and dopamine pathways, positively modulating food reward mechanisms. Regarding appetite-regulating hormones, resistance training suppresses ghrelin and increases peptide YY (PYY), while aerobic training improves leptin sensitivity. High-intensity interval training (HIIT) has shown particular efficacy in reducing visceral adiposity in postmenopausal women. Combined exercise programs integrating both aerobic and resistance components appear most effective for improving overall body composition [19].

### 6.4. Emerging Therapeutic Approaches

Novel therapeutic strategies targeting specific molecular mechanisms of appetite dysregulation during menopause are under investigation. Selective estrogen receptor modulators (SERMs) provide tissue-selective ER activation, potentially offering metabolic benefits while minimizing risks associated with systemic estrogen therapy. Given the importance of the melanocortin system, pharmacological activation of MC4R represents a potential therapeutic approach, as estrogen synthesized in the central nervous system enhances MC4R expression and reduces food intake. Microbiome-targeted interventions, including prebiotics, probiotics, and synbiotics, represent promising approaches to improve metabolic outcomes in postmenopausal women. Integrating multiple therapeutic modalities—dietary modification, physical activity, behavioral interventions, and pharmacological therapies—is likely to provide optimal outcomes for managing eating behavior changes and metabolic dysfunction during menopause. The main therapeutic strategies for managing eating behavior and metabolic changes during the menopausal transition are summarized in Table 2.

## 7. Discussion

This narrative review synthesizes current evidence on the molecular mechanisms, clinical manifestations, and therapeutic approaches related to eating behavior changes in menopausal women. The findings reveal a complex, multi-level cascade of changes initiated by estrogen deficiency that profoundly affects appetite regulation, food intake patterns, body composition, and metabolic health.

At the molecular level, estrogen deficiency disrupts hypothalamic circuits regulating energy homeostasis through multiple mechanisms. The decline in ERα signaling in key hypothalamic nuclei leads to decreased activity of anorexigenic POMC neurons and increased activity of orexigenic NPY/AgRP neurons, shifting the balance toward increased appetite and reduced satiety [5,6,12]. Peripheral hormonal signals, including leptin resistance and altered ghrelin dynamics, further contribute to appetite dysregulation [3,6,20]. Neurotransmitter systems, particularly serotonin and dopamine, are also affected, influencing both homeostatic and hedonic aspects of eating [13,14,21].

These molecular changes translate into clinically significant alterations in eating behavior. Postmenopausal women experience increased hunger, enhanced desire to eat, and decreased satiety, leading to hyperphagia and preferential consumption of high-fat and high-sugar foods [3]. The risk of disordered eating behaviors, including binge eating, increases during the menopausal transition [10,11]. These behavioral changes contribute to weight gain averaging 2–3 kg, with concerning redistribution of fat from subcutaneous to visceral depots [3,6,17].

Substantial increases in visceral adiposity and a tripling of metabolic syndrome prevalence are observed in postmenopausal women. The metabolic consequences of these changes are substantial. Substantial increases in visceral adiposity and a tripling of metabolic syndrome prevalence are observed in postmenopausal women [5,6]. The combination of increased visceral fat, insulin resistance, dyslipidemia, and hypertension creates a high-risk cardiometabolic profile that contributes to a 26–30% increase in coronary heart disease mortality. Emerging evidence implicates gut microbiota alterations in menopausal metabolic changes, opening new therapeutic avenues [18]. Therapeutic strategies must address multiple levels: HRT effectively restores estrogen signaling [4,14,17]; nutritional interventions improve metabolic parameters [17,25]; lifestyle modifications are essential [22]; and emerging molecular and microbiome-targeted approaches hold promise for future development.

Important gaps in current knowledge include: heterogeneity in individual responses suggesting genetic or epigenetic modulation of susceptibility; incompletely defined optimal timing, formulation, and duration of HRT; mechanisms linking gut microbiota alterations to eating behavior changes; and the need for rigorous clinical trials of emerging approaches. Limitations of the current evidence base include the predominance of cross-sectional studies, limited longitudinal follow-up, predominance of animal mechanistic studies, failure to distinguish between natural and surgical or iatrogenic menopause, and limited representation of ethnic and cultural diversity in study populations. Despite these limitations, the accumulated evidence provides a robust framework demonstrating that menopausal metabolic dysfunction is not inevitable but is a specific consequence of estrogen deficiency that can be prevented or mitigated through targeted interventions.

## 8. Conclusions

The menopausal transition represents a critical period characterized by profound changes in eating behavior driven by estrogen deficiency-induced alterations in hypothalamic appetite regulation, peripheral hormonal signaling, and neurotransmitter systems. At the molecular level, declining estradiol disrupts the balance between orexigenic and anorexigenic neuropeptide systems, induces leptin resistance, alters ghrelin dynamics, and reduces serotonergic and dopaminergic tone. These molecular changes manifest clinically as increased appetite, enhanced food reward sensitivity, altered macronutrient preferences, and elevated risk of disordered eating behaviors.

The consequences extend beyond weight gain to include preferential visceral fat accumulation, sarcopenia, insulin resistance, and metabolic syndrome, creating a high-risk cardiometabolic profile. The 44% increase in visceral adiposity and threefold increase in metabolic syndrome prevalence observed in postmenopausal women underscore the clinical significance of these changes. Emerging evidence implicating gut microbiota alterations adds another layer of complexity to this multifaceted syndrome.

Effective management requires a comprehensive, multi-level approach integrating hormone replacement therapy (when appropriately indicated), nutritional interventions, lifestyle modifications, and emerging molecular or microbiome-targeted strategies. Future research should focus on identifying factors that modulate individual susceptibility, optimizing therapeutic interventions, elucidating gut microbiota–eating behavior relationships, and conducting rigorous clinical trials. By integrating molecular insights with clinical practice, we can improve metabolic health and quality of life for the growing population of postmenopausal women worldwide.

## Figures and Tables

**Figure 1:**
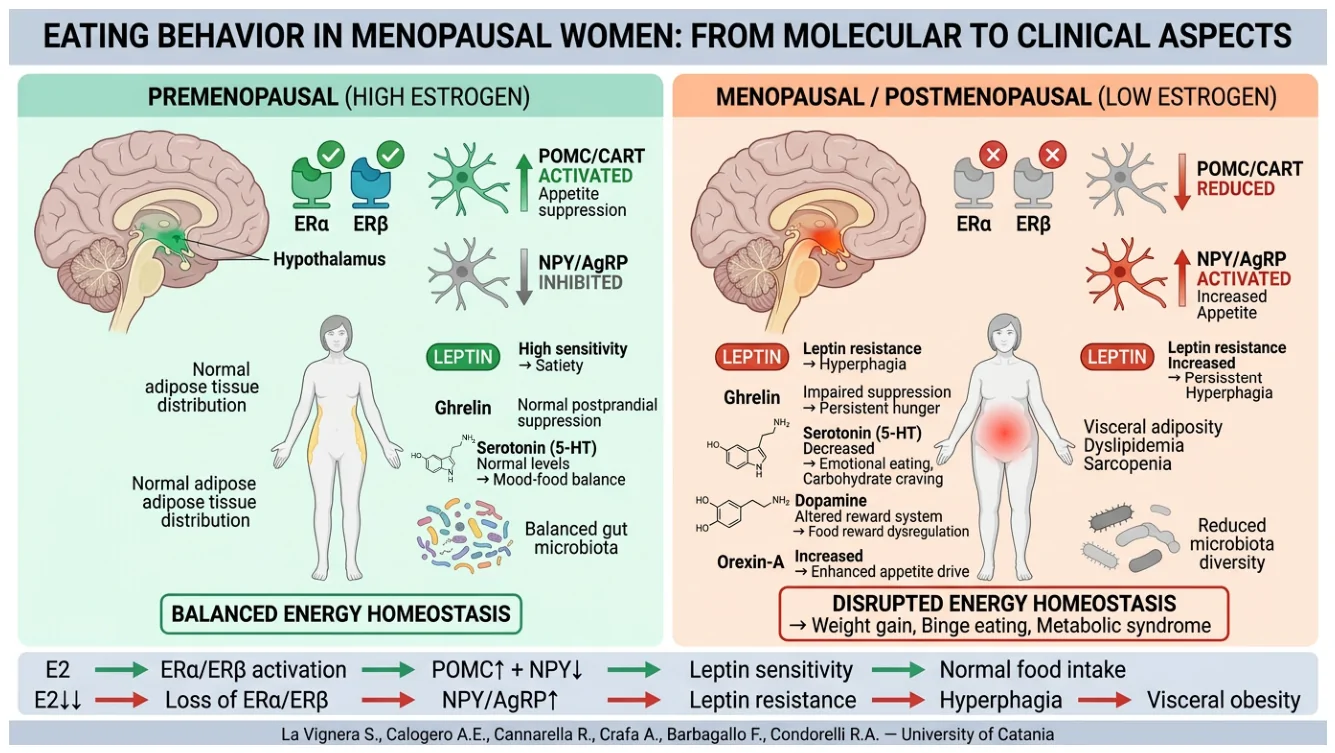
Schematic representation of the principal molecular and clinical changes in eating behavior during the menopausal transition. Estrogen deficiency disrupts hypothalamic circuits (ERα/ERβ signaling, NPY/AgRP/POMC balance), peripheral hormone regulation (leptin resistance, increased ghrelin), and neurotransmitter pathways (serotonin, dopamine), collectively resulting in increased appetite, hedonic eating, visceral adiposity, dyslipidemia, and sarcopenia. HRT: hormone replacement therapy.

**Table 1:** Summary of key molecular and hormonal changes affecting eating behavior during the menopausal transition.

Mediator	Change in Menopause	Effect on Eating Behavior	Molecular Mechanism
Estradiol (E2)	↓ Decreased	↑ Appetite; ↓ satiety	Reduced ERα/ERβ signaling in hypothalamic nuclei (ARC, VMH)
Neuropeptide Y (NPY)	↑ Increased activity	↑ Food intake (orexigenic)	Loss of ERα-mediated suppression of NPY gene expression
Agouti-related peptide (AgRP)	↑ Increased activity	↑ Food intake; MC4R antagonism	Co-expressed with NPY; inhibits melanocortin signaling
POMC/α-MSH	↓ Decreased	↓ Satiety signaling	Reduced ERα-mediated POMC transcription in ARC neurons
Leptin	↑ Increased (with resistance)	↑ Appetite despite elevated leptin levels	Impaired hypothalamic leptin receptor signaling; leptin resistance
Ghrelin	↓ Reduced postprandial suppression	Impaired satiety; altered meal patterns	Reduced postprandial ghrelin suppression; altered gastric emptying
Serotonin	↓ Decreased	↑ Carbohydrate cravings; ↑ hedonic eating	Reduced estrogen-dependent serotonin synthesis and reuptake inhibition
Dopamine	↓ Decreased mesolimbic signaling	↑ Food reward sensitivity; ↑ hyperphagia	Reduced D2 receptor signaling in nucleus accumbens pathways
Orexin-A	↑ Increased	↑ Appetite stimulation; altered energy balance	Hypothalamic orexin upregulation following estrogen withdrawal

**Table 2:** Overview of therapeutic strategies for managing eating behavior alterations and metabolic changes in menopausal women. Evidence level criteria: High = consistent evidence from multiple RCTs or meta-analyses; Moderate–High = evidence from RCTs and prospective cohort studies; Moderate = evidence from controlled trials or consistent observational data; Emerging = preliminary evidence from pilot studies or mechanistic research requiring confirmation.

Therapeutic Strategy	Mechanism of Action	Key Benefits	Evidence Level
Hormone Replacement Therapy (HRT)	Restores estrogen signaling; normalizes NPY/POMC balance; improves leptin sensitivity and serotonin activity	Reduces appetite dysregulation; attenuates visceral fat accumulation; improves insulin sensitivity	Moderate–High
Mediterranean Diet	Anti-inflammatory dietary pattern; modulates gut microbiota; improves insulin sensitivity	Reduces BMI, visceral adiposity, and metabolic syndrome risk	Moderate–High
High-protein diet	Enhances satiety via GLP-1 and PYY release; preserves lean muscle mass	Reduces binge eating risk; attenuates sarcopenia progression	Moderate
Calcium and Vitamin D supplementation	Supports bone metabolism; modulates adipogenesis and body composition	Reduces osteoporosis risk; improves metabolic parameters	Moderate
Resistance exercise	Maintains lean mass; improves insulin sensitivity; modulates ghrelin levels	Reduces visceral adiposity; counteracts sarcopenia; improves metabolic markers	High
Aerobic exercise	Increases energy expenditure; modulates serotonin and dopamine; reduces stress eating	Weight management; improves mood; reduces emotional eating	High
Mindfulness-based interventions	Reduces emotional and binge eating; modulates stress-HPA axis activity	Reduces stress-related and binge eating behaviors; improves quality of life	Moderate
GLP-1 receptor agonists (e.g., semaglutide)	Mimics postprandial GLP-1 signaling; enhances satiety; reduces appetite	Weight reduction; improvement in metabolic and cardiometabolic markers	Emerging
Probiotics/Prebiotics	Restores estrobolome diversity; reduces LPS-mediated inflammation	Improves gut microbiota composition; modulates metabolic markers	Emerging

## Data Availability

No new data were created or analyzed in this study. Data sharing is not applicable to this article.
